# Novel Small-Molecule Scaffolds as Candidates against the SARS Coronavirus 2 Main Protease: A Fragment-Guided in Silico Approach

**DOI:** 10.3390/molecules25235501

**Published:** 2020-11-24

**Authors:** Teresa L. Augustin, Roxanna Hajbabaie, Matthew T. Harper, Taufiq Rahman

**Affiliations:** Department of Pharmacology, University of Cambridge, Tennis Court Road, Cambridge CB2 1PD, UK; ta451@cam.ac.uk (T.L.A.); rh731@cam.ac.uk (R.H.); mth29@cam.ac.uk (M.T.H.)

**Keywords:** coronavirus, COVID-19, SARS-CoV-2 M^pro^, main protease, fragment-guided, drug discovery, pharmacophore, molecular docking, natural products, in silico

## Abstract

The ongoing pandemic caused by the novel coronavirus has been the greatest global health crisis since the Spanish flu pandemic of 1918. Thus far, severe acute respiratory syndrome coronavirus 2 (SARS-CoV-2) has resulted in over 1 million deaths, and there is no cure or vaccine to date. The recently solved crystal structure of the SARS-CoV-2 main protease has been a major focus for drug-discovery efforts. Here, we present a fragment-guided approach using ZINCPharmer, where 17 active fragments known to bind to the catalytic centre of the SARS-CoV-2 main protease (SARS-CoV-2 M^pro^) were used as pharmacophore queries to search the ZINC databases of natural compounds and natural derivatives. This search yielded 134 hits that were then subjected to multiple rounds of in silico analyses, including blind and focused docking against the 3D structure of the main protease. We scrutinised the poses, scores, and protein–ligand interactions of 15 hits and selected 7. The scaffolds of the seven hits were structurally distinct from known inhibitor scaffolds, thus indicating scaffold novelty. Our work presents several novel scaffolds as potential candidates for experimental validation against SARS-CoV-2 M^pro^.

## 1. Introduction

Since the sudden outbreak of novel severe acute respiratory syndrome-coronavirus-2 (SARS-CoV-2) in Wuhan, China in December 2019 [1,2], the resulting pandemic has seriously challenged global healthcare systems and caused more than 1 million deaths (as of 30 September 2020) [3]. As cases have reached a global scale, the disease has become a major disruption to people’s daily lives and the economy [4]. Coronavirus disease 2019 (COVID-19) can result in pneumonia and respiratory failure that can be fatal [5], especially in the presence of other comorbidity factors. Despite ongoing efforts by several academic and industrial groups towards developing vaccines against SARS-CoV-2 [6], we are yet to have any vaccine approved by the Food and Drug Administration (FDA).

**Figure 1:**
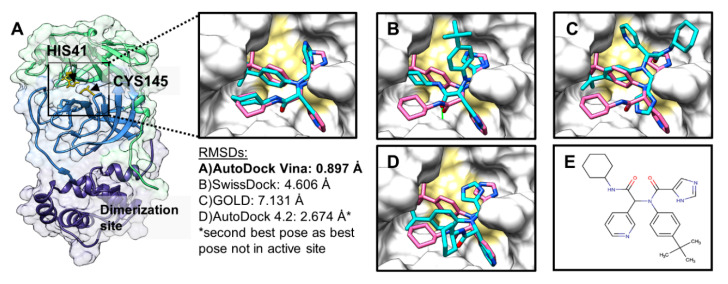
Representative top-ranked poses of noncovalent inhibitor X77 from multiple docking programs (cyan sticks) and original cocrystallised pose of X77 (pink sticks) against severe acute respiratory syndrome coronavirus 2 (SARS-CoV-2) main protease (M^pro^) (Protein Data Bank (PDB) ID: 6W63) with root-mean-square-deviation (RMSD) values. Catalytic residues, histidine 41 (His41), and cysteine 145 (Cys145) highlighted in gold. (**A**) Cartoon and surface representation of SARS-CoV-2 M^pro^ crystal structure (PDB ID: 6W63) with three domains labelled (Domain I: green; Domain II: blue; Domain III: purple). Zoom in shows AutoDock Vina result; (**B**) SwissDock result; (**C**) GOLD result; (**D**) AutoDock 4.2 result; (**E**) 2D structure of X77.

During the life cycle of SARS-CoV-2, two large polyproteins are translated and subsequently cleaved by two viral proteases, main protease SARS-CoV-2 3CL M^pro^ (more simply, SARS-CoV-2 M^pro^) and a papain-like protease (PL^pro^) [7]. The dependence of the virus on M^pro^, and given that there are no similar human proteases, make this protein a promising drug target [8,9,10]. SARS-CoV-2 M^pro^ consists of three domains. Domains I and II comprise β-barrels forming a chymotrypsin structure and harbouring catalytic dyad histidine 41 (His41), and cysteine 145 (Cys145) [11]. Domain III consists of α-helices (Figure 1A) [12]. For its catalytic activity, M^pro^ needs to dimerise, forming contacts with both the N- and C-terminal domains of the other protomer [13]. SARS-CoV-2 M^pro^ shows 96% sequence similarity with SARS-CoV-1 M^pro^, and the root-mean-square deviation (RMSD) between the two free enzyme structures is 0.53 Å for all Cα positions [8]. The strong sequence and structural similarity suggest that M^pro^ inhibitors developed for SARS-CoV-1 could also be effective against SARS-CoV-2, although none of these inhibitors is an approved treatment so far. 

Drug-discovery efforts against SARS-CoV-1 M^pro^ have primarily involved finding peptidic and small-molecule inhibitors targeting the active site [12,14]. Peptidic inhibitors contain chemical warhead groups, such as Michael acceptors, to form a covalent bond with a specific cysteine residue (Cys145) within the active site [12]. The peptidic inhibitor, called N3, (half-maximal inhibitory concentration (IC_50_) = 16.77 μM) was the first to be cocrystallised with SARS-CoV-2 M^pro^ [14]. Several other peptidic and nonpeptidic covalent inhibitors have since been described [15], although none has been approved as a cure for COVID-19 to date. On the other hand, noncovalently interacting small molecules may produce fewer off-target side effects and toxicity [16]; therefore, our work focuses on these. The most potent noncovalent inhibitor of SARS-CoV-1 M^pro^ so far is ML188 (antiviral IC_50_ = 12.9 μM) [7,16,17], and it has also been suggested as a broad-spectrum inhibitor of coronavirus M^pro^s [18]. To date, there is only one SARS-CoV-2 M^pro^ structure (Protein Data Bank (PDB) ID: 6W63) with a cocrystallised noncovalent inhibitor (X77) [19]. Interestingly, X77 is similar in structure to potent noncovalent SARS-CoV-1 M^pro^ inhibitor ML188.

Given the urgency for developing effective drugs against COVID-19, Diamond Light Source, UK (www.diamond.ac.uk) performed a crystallographic fragment screening campaign (XChem fragment screen) [20] against SARS-CoV-2 M^pro^. Of these fragments, 23 were found to be noncovalently bound to the active site of SARS-CoV-2 M^pro^, 17 of which bound with high confidence. On the basis of these noncovalently bound fragments, our study aimed to identify novel scaffolds from natural products and derivatives against the active site of SARS-CoV-2 M^pro^. In total, 134 natural products and their derivatives were obtained through a ZINCPharmer [21] pharmacophore search and subjected to multiple rounds of molecular docking against SARS-CoV-2 M^pro^ to evaluate avidity for binding in silico. Docking scores and ligand–protein interactions were compared to those of known noncovalent inhibitor X77. This in silico fragment-guided approach eventually resulted in the identification of seven novel chemical scaffolds that could be taken further to in vitro testing against SARS-CoV-2 M^pro^. 

## 2. Results

### 2.1. Identification of Initial Hits through Fragment-Derived Pharmacophore-Based Screening

Virtual screening in ZINCPharmer with pharmacophore queries from the 17 fragments known to noncovalently bind to the active centre of SARS-CoV-2 M^pro^ [20] led to the identification of 134 scaffolds belonging to ZINC natural products and their derivative subset. The ZINC library was used as it is a rich source of diverse and often bioactive chemical scaffolds that are commercially available. The 134 obtained scaffolds were later subjected to blind and focused docking against the M^pro^ structure. 

### 2.2. Docking-Protocol Validations

Before docking the 134 hits from the ZINCPharmer-based screening, we sought out to evaluate the accuracy and reproducibility of our docking protocol by performing a validation or self-docking exercise with AutoDock Vina [22], AutoDock 4.2 [23,24], SwissDock [25], and GOLD [26]. For this, known inhibitor X77 was blindly (pseudoblindly for GOLD) docked against M^pro^, and poses were ranked on the basis of the default scoring functions of these algorithms. As shown in Figure 1, AutoDock Vina, in blind docking mode with exhaustiveness of 24, could almost retrieve the original pose of X77, unlike AutoDock 4.2, GOLD, or SwissDock. The docked pose of AutoDock Vina was very closely superimposed onto the original pose within the active site of M^pro^ with an RMSD of ~0.9 Å, which was well within the acceptable range (≤2 Å) for successful validation of self-docking [27]. Moreover, docked poses and relevant scores (ΔG = −8.52 kcal/mol, *n* = 5) for X77 were highly reproducible in AutoDock Vina when carried out in 5 independent runs (standard error: 0.06 kcal/mol). Therefore, we used AutoDock Vina for all our blind and focused docking, with exhaustiveness set to 24.

### 2.3. Evaluation of ZINCPharmer Hits Using Molecular Docking

**Figure 2:**
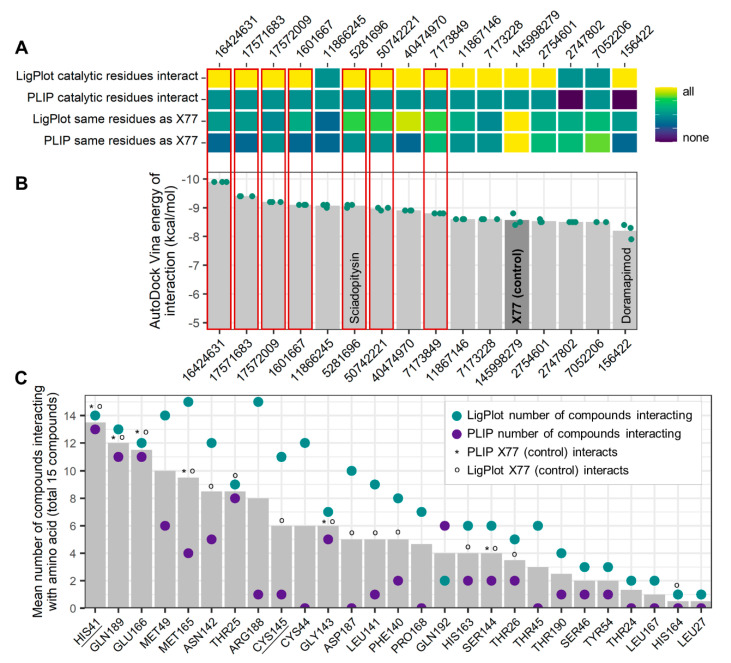
Overview of AutoDock Vina scores obtained in focused docking and protein–ligand interactions of a selection of compounds. Compound labels are PubChem compound IDs (CIDs) with available names shown on the bar. (**A**) Analysis of protein–ligand interactions using Protein–Ligand Interaction Profiler (PLIP) and LigPlot^+^ focusing on similar interactions as X77 original cocrystallised control and interactions with catalytic dyad; (**B**) AutoDock Vina mean predicted energy of interaction scores from three independent runs; (**C**) frequency of compounds interacting with residues in PLIP and LigPlot^+^. Catalytic residues, histidine 41 (His41), and cysteine 145 (Cys145) are underlined.

All initial 134 compounds from ZINCPharmer were subjected to blind docking against monomeric SARS-CoV-2 M^pro^. In the blind-docking round, the top-ranked (i.e., with the lowest ΔG) poses of most compounds, except for PubChem compound ID (CID) 2754601 (Supporting Information, Table S1), were in the active site of the protease. Out of the 134 molecules, only 15 scaffolds (Supporting Information, Figure S2) had ΔG values comparable to or lower than that of X77 (ΔG ~8.5 kcal/mol), which we arbitrarily used as a cut-off. Those 15 molecules were then subjected to possible pose refinement through focused docking (Figure 2). The achieved scores in the blind and focused docking of the chosen 15 molecules were very similar regardless of grid-size differences (Supporting Information, Table S1 and Figure 2).

**Figure 3:**
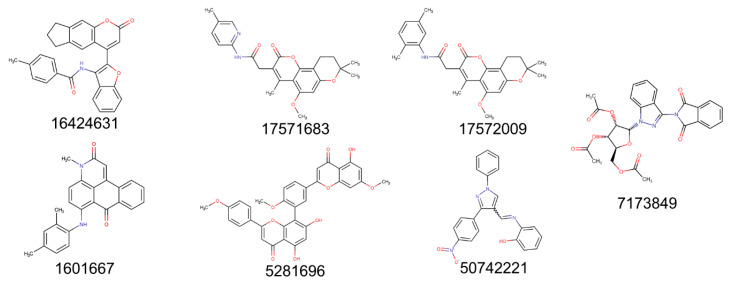
Two-dimensional structures of top seven selected compounds labelled with PubChem CIDs.

Out of the 15 evaluated molecules in focused docking, the top 7 hits (Figure 3) were selected (highlighted red in Figure 2A) on the basis of their ΔG values and the nature of interactions with the protease, and successful blind docking against the active site of the dimeric M^pro^ (results not shown). All these compounds had reproducible scores and poses in each of the 3 independent docking runs. PubChem CID 16424631 had the lowest mean value of −9.9 kcal/mol, followed by PubChem CIDs 17571683 and 17572009, with scores of −9.4 and −9.2 kcal/mol, respectively. All hits are commercially available (Supporting Information, Table S2). While we tried to ensure that there was diversity in the chosen set of scaffolds, PubChem CIDs 17571683 and 17572009 have very similar scaffolds, which led to similar AutoDock Vina scores. Upon visual inspection, none of our top seven selected scaffolds was deemed comparable to any of the known inhibitors of SARS-CoV-1/SARS-CoV-2 M^pro^ listed in the IUPHAR/BPS Guide to Pharmacology [28] and PostEra’s COVID Moonshot page (https://covid.postera.ai/covid/structures) [29]. Overall, 31 unique scaffolds were compared (Supporting Information, Table S3). When searching the ChEMBL database [30], only PubChem CID 5281696 was flagged up as a 100% match with sciadopitysin, while the other 6 molecules as queries produced no hits. Interestingly, sciadopitysin (PubChem CID 5281696) was previously found to inhibit SARS-CoV-1 M^pro^ in a fluorescence-resonance energy-transfer (FRET) assay (38.4 ± 0.2 μM) [31], demonstrating the predictive power of our approach.

### 2.4. Protein–Ligand Interactions

**Figure 4:**
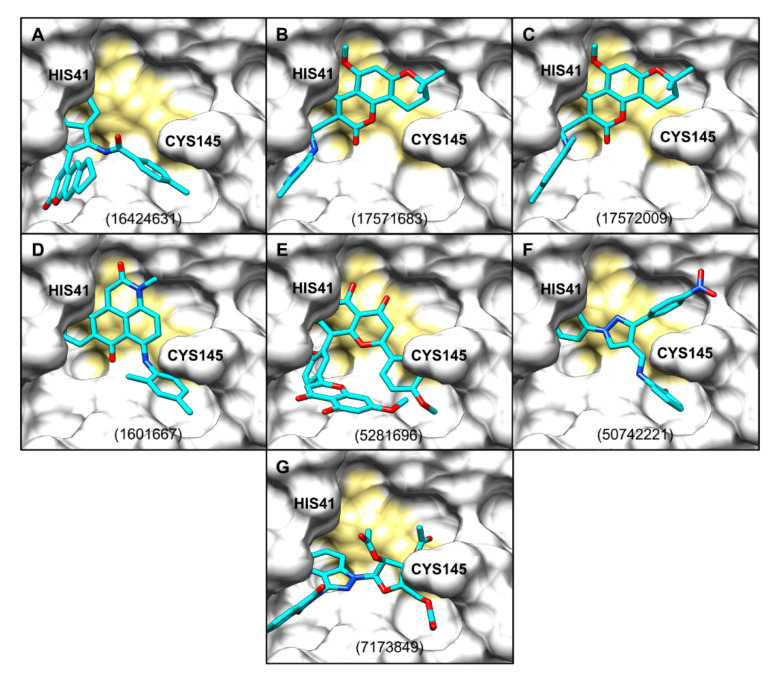
(**A**–**G**). AutoDock Vina poses (cyan sticks) from focused docking against active site of SARS-CoV-2 M^pro^, shown as white surface. Catalytic dyad residues highlighted in gold. Compound labels are PubChem CIDs.

**Figure 5:**
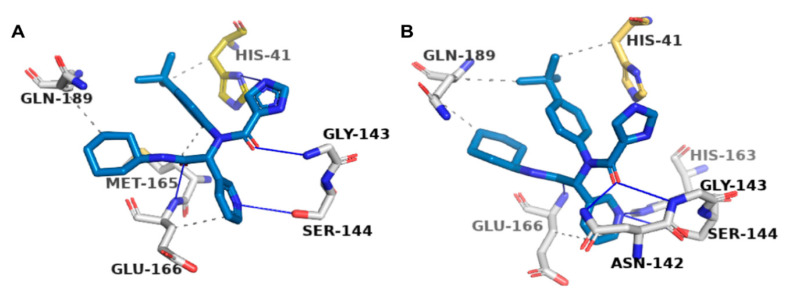
Predicted interactions in PLIP of X77 ligand (steel blue) on SARS-CoV-2 M^pro^ (PDB ID: 6W63, white). (**A**) Original cocrystallised X77 pose; (**B**) X77 pose from AutoDock Vina. Hydrogen bonds shown as blue lines, and hydrophobic interactions shown as dotted grey lines. Catalytic residue histidine 41 (His-41) highlighted in gold.

**Figure 6:**
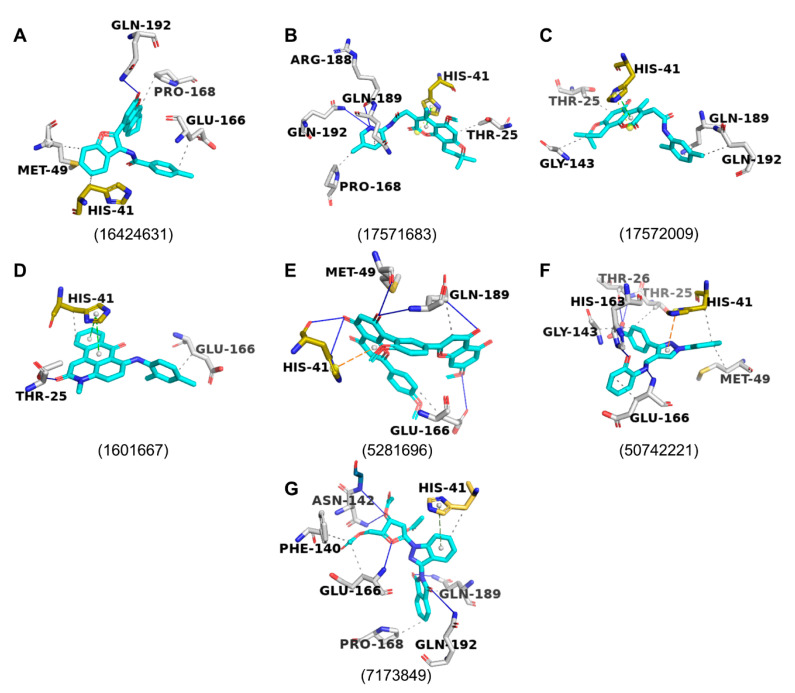
(**A**–**G**). Predicted interactions in PLIP of docked poses of top seven selected compounds (cyan sticks) with SARS-CoV-2 M^pro^ (PDB ID: 6W63, white sticks). Compound labels are PubChem CIDs. Hydrogen bonds (blue lines), hydrophobic interactions (dotted grey lines), π–π stacking interactions (dashed green lines), π–cation interactions (dashed orange lines), and salt bridges (dotted yellow line) are shown. Catalytic dyad, His-41, and Cys-145 highlighted in gold.

All seven chosen molecules seemed to span multiple subpockets within the active site of SARS-CoV-2 M^pro^ (Figure 4). The poses were also analysed for interactions with the M^pro^ in Protein–Ligand Interaction Profiler (PLIP; Figure 5 and Figure 6) and LigPlot^+^ (Supporting Information, Figures S3 and S4), and are compared in Figure 2A. PLIP and LigPlot^+^ are based on different algorithms, with PLIP predicting interactions in 3D and LigPlot^+^ in 2D. Therefore, the two programmes are not directly comparable, but together they serve the purpose of providing a more holistic picture on potential protein–ligand interactions. In both PLIP and LigPlot^+^, most compounds interacted with at least one of the two catalytic residues (His41 and Cys145; Figure 2A) via hydrophobic interactions or hydrogen bonds. The known inhibitor, X77, also only showed interaction with His41 in PLIP (Figure 5), while both His41 and Cys145 were shown to interact in LigPlot^+^ (Supporting Information, Figure S3). Generally, there appeared to be more predicted interactions in LigPlot^+^, which were primarily hydrophobic, whereas PLIP seemed to highlight more hydrogen bonds, fewer hydrophobic interactions, and overall fewer interactions (Figure 6 and Supporting Information, Figure S4). Therefore, it is not surprising that fewer compounds were shown to interact with both catalytic residues in PLIP, while most compounds did in the predictions from LigPlot^+^. 

Besides comparing the predicted interactions with the catalytic dyad between the compounds, we compared interacting residues with those known for X77 (Figure 2A). None of the compounds had the same predicted interactions as the X77 control (Figure 5 and Supporting Information, Figure S3), but PubChem CIDs 5074221, 5281696 (sciadopitysin), 7173849, and 7052206 showed the greatest similarity to X77 in PLIP and LigPlot^+^-based analyses (Figure 2A).

Of the 15 molecules that were chosen for focused docking, more than 76% in their top-ranked docked poses interacted with His41, Glu166, and Gln189 (Figure 2C and Figure 6). Such interactions were also observed for X77 in the original crystallographically obtained pose (Figure 2C and Figure 5, and Supporting Information, Figure S3).

### 2.5. Ligand Properties

The predicted properties of the ligands are presented in Table 1. Four of the seven compounds comply with Lipinski’s rule of five (PubChem CIDs: 17571683, 17572009, 1601667, and 50742221). One of the compounds (PubChem CID: 50742221) had an alert for having a pan-assay interference compound (PAINS) feature. Six of the compounds were predicted to contain toxic groups, (PubChem CIDs: 16424631, 17571683, 17572009, 1601667, 50742221, and 7173849), also known as Brenk alerts [32]. Three compounds were predicted to have moderate solubility (PubChem CIDs: 17571683, 17572009, 1601667), while the others were predicted to have poor solubility. Most of the compounds were predicted to theoretically have good oral pharmacokinetics (high gastrointestinal absorption), except for PubChem CIDs 5281696 and 7173849. 

## 3. Discussion

Amid the ongoing pandemic, there are currently no licensed antiviral medications to cure COVID-19 or an approved vaccine to prevent the infection. Given this, there has been an immense focus on identifying compounds for testing against the coronavirus’ main protease [38,39]. In silico drug-discovery tools have been widely used to expedite this process [40,41]. Here, we designed and implemented a unique fragment-guided pharmacophore-based in silico approach. A dual combination of fragment-derived pharmacophore-based screening and molecular docking was used. We presented seven novel scaffolds as potential candidates for prospective experimental validation against the M^pro^ (Figure 3). We deliberately considered libraries of purchasable natural products and their derivatives as an important source of diverse bioactive chemical scaffolds, many of which were utilised as chemical probes, drugs, or leads for developing modern drugs that notably include antimicrobials [42,43]. While we have not yet found a use for any method similar to ours in identifying novel chemotypes to test against SARS-CoV-2 M^pro^, one of our top seven hits (sciadopitysin) was also identified in a recent in silico study [44]. Interestingly, sciadopitysin was previously reported to inhibit SARS-CoV-1 M^pro^ in vitro with an IC_50_ of 38.4 µM [31]. 

Previous in silico work mainly focused on identifying already approved drugs that can be repurposed against the M^pro^ [45,46,47,48,49,50,51,52]. By now, this in silico approach has been widely exhausted, and there are hundreds of hits reported in the literature. Although we acknowledge that drug repurposing is a highly sought solution given the urgency of the pandemic, we recognise the merit of exploring a much larger drug or leadlike chemical space for finding novel hits or leads toward specifically designing potent antiviral agents against the novel coronavirus’ M^pro^. 

The identification of diverse scaffolds with inhibitory activity against the M^pro^ can facilitate the drug-discovery process through scaffold hopping [53,54]. The greater the number of diverse scaffolds tested against the M^pro^ is, the more functional data are available to delineate which key ligand groups and protein amino acids play an important role in ligand–protein binding, and the inhibition of protein activity [53,55]. For example, the COVID Moonshot Initiative [29] has invited scientists from around the world to submit structures for in vitro testing to use structure–activity relationships and identify the most potent scaffolds. For any drug-discovery venture, several distinct chemical classes are always needed with which to begin, since there is often attrition in the number of scaffolds as they move from various stages of preclinical development. We confirmed the scaffold novelty of our set by taking two approaches: the visual assessment of known SARS-CoV-1/SARS-CoV-2 M^pro^ inhibitors, and a more objective automated search of the ChEMBL database and the PostEra COVID Moonshot platform. 

Inhibitory fragments used in this work were previously shown to target multiple subpockets within the active M^pro^ site [20]. Therefore, their pharmacophore features were valuable in identifying hits. We used the ZINCPharmer tool, which was successfully used to discover novel inhibitors that are active in vitro [56,57,58,59]. Our integrative approach of combining the predictions of two ligand–protein interaction predictors (PLIP [60] and LigPlot^+^ [61]) also allowed for us to assess whether the predicted binding affinity scores for the compounds were reflecting key inhibitory residues in the active site. Most tested compounds were predicted to interact with at least one of the catalytic residues in PLIP and LigPlot^+^, and had similar interactions to the chosen X77 control ligand. LigPlot^+^ assesses interactions in 2D, while PLIP does so in 3D, including more interactions apart from hydrogen bonds and hydrophobic interactions (salt bridges, π–π stacking, and π–cation interactions). This may grant PLIP greater reliability. Yoshino et al. (2020) [62] found that His41, Gly143, and Glu166 were important residues for peptide inhibitors in molecular-dynamics simulations. Our control molecule, X77, interacted with all three of these residues, while His41 and Glu166 were the two most common interacting residues within our compound set (Figure 2C). 

Our study highlights a novel set of seven commercially available compounds with favourable predicted free energy of interactions and likely poses against SARS-CoV-2 M^pro^. We propose that these molecules represent potential candidates for prospective in vitro validation against SARS-CoV-2 M^pro^. While our study is solely based on in silico analyses, multiple examples show that AutoDock Vina scores are correlated with experimental data [63,64,65,66]. If proven to bind to and inhibit the protease, our scaffolds can be subjected to further optimisation through the iterative use of medicinal chemistry, and further testing against the M^pro^ to evaluate their effects on its activity. This could significantly improve their potency, safety, and stability. We also present an in silico pipeline that can be applied to screen a much larger drug or leadlike chemical space, and this could lead to the identification of more novel scaffolds to be tested against SARS-CoV-2 M^pro^. 

## 4. Materials and Methods 

### 4.1. Virtual Screening with Fragment-Based Pharmacophores

**Figure 7:**
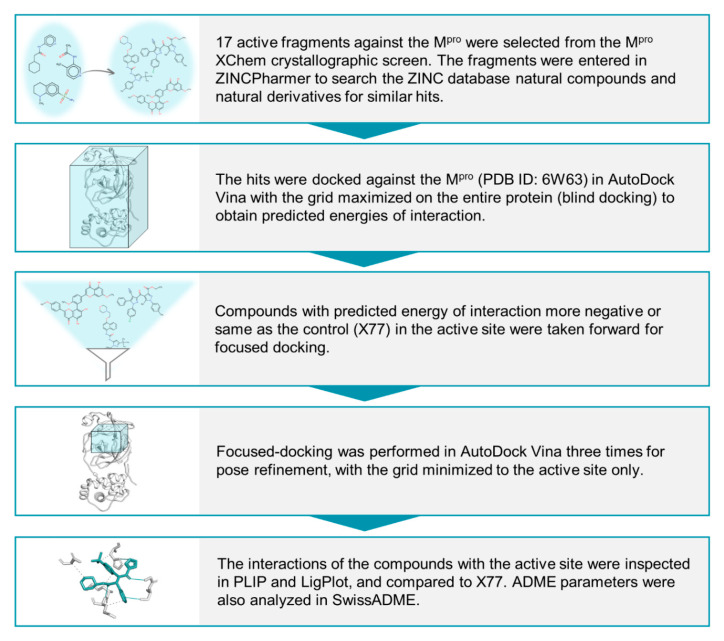
In silico workflow in the present study to identify novel chemical scaffolds from natural products and derivatives that could be experimentally tested against SARS-CoV-2 M^pro^.

The 3D structures of 17 active fragments (Supporting Information, Figure S1) that were cocrystallised with SARS-CoV-2 M^pro^ as noncovalent binders to its active site in Diamond Light Source M^pro^ XChem Screen [20] were obtained from the PDB. Each fragment was entered as a query into ZINCPharmer [21] to screen a subset of the ZINC database comprising natural products and their derivatives [67] in order to identify molecules with similar pharmacophore features (Figure 7). The pharmacophore properties of the fragments were largely kept as the original. However, to maximise the chance of finding more hits, some pharmacophore features deemed nonessential for interacting with the M^pro^ active site were removed. 

### 4.2. Protein-Structure Preparation

The 3D structure of SARS-CoV-2 M^pro^ with cocrystallised noncovalent inhibitor X77 was obtained from PDB (PDB ID: 6W63) [19]. Preparations of the protein for docking were made in ICM-Pro 3.8 [68], including the removal of ligand and water molecules, and the addition of hydrogens.

### 4.3. Molecular Docking

Before performing any docking with the ZINCPharmer-derived hits, validation docking was performed with known inhibitor X77. The latter was blindly docked [69,70] against the entire SARS-CoV-2 M^pro^ structure using AutoDock Vina [22]. The highest-ranked (i.e., with the lowest predicted free energy of interaction, ΔG, kcal/mol) docked pose of X77 was superimposed on its original pose present in the crystal structure, and the RMSD was calculated in DockRMSD v1.1 [71]. The average ΔG value for X77 was calculated out of 5 independent docking runs. The exhaustiveness value for docking was set to 24.

In addition to AutoDock Vina, three other docking programs were initially tested for the blind docking of the X77 control. These programmes included AutoDock 4.2 (AD 4.2) [23,24], GOLD suite version 5.8.0 (CCDC, Cambridge, UK) [26], and the SwissDock server (www.swissdock.ch) [25]. For AD 4.2-based blind docking, the Lamarckian genetic algorithm was used with a grid box that had the same dimensions as those for blind docking with AutoDock Vina. Additionally, pseudoblind docking was performed in GOLD with a larger grid box of 10 Å (instead of the default 6 Å) set around the centre of mass of the X77 control in the active site. For SwissDock, the blind mode was opted for [25].

All molecules derived from the ZINCPharmer-based virtual screening were initially subjected to blind docking against the unliganded monomeric M^pro^ structure using AutoDock Vina. Molecules for docking were obtained from PubChem (https://pubchem.ncbi.nlm.nih.gov) in 3D file format. Following this unbiased docking approach, subsequent focused docking with a grid centred on the active site of the M^pro^ structure was used for a subset of initial hits, the best poses of which had ΔG values comparable to or lower than that of X77. As with blind docking, the exhaustiveness value was set to 24; for each molecule, 3 independent docking runs were performed, and the mean predicted interaction of binding was calculated. Hits scoring well in focused docking were also blindly docked against the dimeric M^pro^ structure to elucidate hits potentially binding to the dimer interface, and only selecting those with predicted binding to the active site. 

### 4.4. Analysis of Protein–Ligand Interactions

Protein–ligand interactions were analysed in the Protein–Ligand Interaction Profiler (PLIP) [60], and LigPlot^+^ 4.5.3 [61]. The resulting interaction profiles were compared with the interactions of known inhibitor X77, and interaction with the catalytic dyad was particularly considered during the selection of hits. 

### 4.5. Scaffold Novelty

For the evaluation of scaffold novelty, the 2D structures of the chosen hits were visually compared against all structures listed on the IUPHAR/BPS Guide to Pharmacology [28] and PostEra’s COVID Moonshot initiative (https://covid.postera.ai/covid/structures) [29] known to be inhibitors of SARS-CoV-1/SARS-CoV-2 M^pro^. PostEra’s COVID Moonshot initiative platform and the entire ChEMBL database (www.ebi.ac.uk/chembl) [30] were also searched to check for scaffold novelty. Where available, bioassay results were checked on PubChem profiles to explore whether a particular compound had previously been tested against the M^pro^.

### 4.6. Exploring Ligand Properties

The SwissADME [32] server (www.swissadme.ch) was used to predict the pharmacokinetic and toxicological properties of the compounds.

### 4.7. Figure Creation

A range of programmes were used to aid in figure creation. These included PyMOL 2.4 (https://pymol.org/2), UCSF Chimera 1.14 [72], R Studio [73], and Microsoft PowerPoint 2016. The 2D chemical structures of ligands were drawn using MarvinSketch 20.16 (ChemAxon Ltd.) [74].

## Figures and Tables

**Table 1:** SwissADME results of top seven selected compounds labelled with PubChem CIDs.

PubChem CID (also Known as)	Druglikeness	Structural Alerts	Pharmacokinetics ^4^	Water Solubility ^5^
16424631	Meets Lipinski’s rules, except MLOGP ^1^ > 4.15	PAINS ^2^: 0 alerts; Brenk ^3^: 1 alert-coumarin	High gastrointestinal (GI) absorption	Poorly soluble; Ilogp ^6^: 3.65
17571683	Meets Lipinski’s rules	PAINS: 0 alerts; Brenk: 1 alert-coumarin	High GI absorption	Moderately soluble; iLOGP: 3.83
17572009	Meets Lipinski’s rules	PAINS: 0 alerts; Brenk: 1 alert-coumarin	High GI absorption	Moderately soluble; iLOGP: 4.40
1601667	Meets Lipinski’s rules	PAINS: 0 alerts; Brenk: 2 alerts-2 polycyclic aromatic hydrocarbons	High GI absorption	Moderately soluble; iLOGP: 3.66
5281696 (sciadopitysin)	Meets Lipinski’s rules except MW > 500	PAINS: 0 alerts; Brenk: 0 alerts	Low GI absorption	Poorly soluble; iLOGP: 4.65
50742221	Meets Lipinski’s rules	PAINS: 1 alert-imine phenol; Brenk: 3 alerts-imine, nitro group, oxygen–nitrogen single bond	High GI absorption	Poorly soluble; iLOGP: 3.15
7173849	Meets Lipinski’s rules except MW > 500 and hydrogen bond acceptors > 10	PAINS: 0 alerts; Brenk: 2 alerts-more than 2 esters, phthalimide	Low GI absorption	Moderately soluble; iLOGP: 2.85

^1^ Moriguchi octanol–water partition coefficient [33], ^2^ pan-assay interference compounds [34], ^3^ Brenk alerts to toxic groups [32], ^4^ according to BOILED egg method [35], ^5^ predicted using LogS (Ali) [36], ^6^ octanol/water partition coefficient [37].

## Supplementary Materials

The following are available online. Figure S1: 2D structures of 17 active fragments from M^pro^ XChem Screen; Figure S2: 2D structures of tested compounds; Figure S3: X77 LigPlot^+^ interactions with M^pro^; Figure S4: top seven hits of LigPlot^+^ interactions with M^pro^; Table S1: Blind docking scores for all 134 compounds; Table S2: List of vendors, IDs, and SMILES of 15 compounds from focused docking; Table S3: list of known SARS-CoV-1/SARS-CoV-2 M^pro^ inhibitors.

## References

[1] Wu F., Zhao S., Yu B., Chen Y.-M., Wang W., Song Z.-G., Hu Y., Tao Z.-W., Tian J.-H., Pei Y.-Y. (2020). A new coronavirus associated with human respiratory disease in China. Nature.

[2] Coronaviridae Study Group of the International Committee on Taxonomy of Viruses (2020). The species Severe acute respiratory syndrome-related coronavirus: Classifying 2019-nCoV and naming it SARS-CoV-2. Nat. Microbiol..

[3] World Health Organization Coronavirus Disease (COVID-19) Pandemic. https://www.who.int/emergencies/diseases/novel-coronavirus-2019.

[4] McKee M., Stuckler D. (2020). If the world fails to protect the economy, COVID-19 will damage health not just now but also in the future. Nat. Med..

[5] Nutho B., Mahalapbutr P., Hengphasatporn K., Pattaranggoon N.C., Simanon N., Shigeta Y., Hannongbua S., Rungrotmongkol T. (2020). Why Are Lopinavir and Ritonavir Effective against the Newly Emerged Coronavirus 2019? Atomistic Insights into the Inhibitory Mechanisms. Biochemistry.

[6] Krammer F. (2020). SARS-CoV-2 vaccines in development. Nat. Cell Biol..

[7] Macchiagodena M., Pagliai M., Procacci P. (2020). Identification of potential binders of the main protease 3CLpro of the COVID-19 via structure-based ligand design and molecular modeling. Chem. Phys. Lett..

[8] Zhang L., Lin D., Sun X., Curth U., Drosten C., Sauerhering L., Becker S., Rox K., Hilgenfeld R. (2020). Crystal structure of SARS-CoV-2 main protease provides a basis for design of improved α-ketoamide inhibitors. Science.

[9] Yang H., Yang M., Ding Y., Liu Y., Lou Z., Zhou Z., Sun L., Mo L., Ye S., Pang H. (2003). The crystal structures of severe acute respiratory syndrome virus main protease and its complex with an inhibitor. Proc. Natl. Acad. Sci. USA.

[10] Anand K. (2003). Coronavirus Main Proteinase (3CLpro) Structure: Basis for Design of Anti-SARS Drugs. Science.

[11] Ionescu M.I. (2020). An Overview of the Crystallized Structures of the SARS-CoV-2. Protein J..

[12] Pillaiyar T., Manickam M., Namasivayam V., Hayashi Y., Jung S.-H. (2016). An Overview of Severe Acute Respiratory Syndrome–Coronavirus (SARS-CoV) 3CL Protease Inhibitors: Peptidomimetics and Small Molecule Chemotherapy. J. Med. Chem..

[13] Zhong N., Zhang S., Zou P., Chen J., Kang X., Li Z., Liang C., Jin C., Xia B. (2008). Without Its N-Finger, the Main Protease of Severe Acute Respiratory Syndrome Coronavirus Can Form a Novel Dimer through Its C-Terminal Domain. J. Virol..

[14] Jin Z., Du X., Xu Y., Deng Y., Liu M., Zhao Y., Zhang B., Li X., Zhang L., Peng C. (2020). Structure of M(pro) from SARS-CoV-2 and discovery of its inhibitors. Nature.

[15] Reddy V.P., Elkhateeb E., Jo H., Natalie N., Lythgoe E., Tang W., Jamei M., Sharma S., Hodjegan A.R. (2020). Pharmacokinetics under the COVID-19 storm!. Drug Targets Potential Treat. J. Med. Chem..

[16] Jacobs J., Grum-Tokars V., Zhou Y., Turlington M., Saldanha S.A., Chase P., Eggler A., Dawson E.S., Baez-Santos Y.M., Tomar S. (2013). Discovery, Synthesis, and Structure-Based Optimization of a Series of *N*-(tert-Butyl)-2-(*N*-arylamido)-2-(pyridin-3-yl) Acetamides (ML188) as Potent Noncovalent Small Molecule Inhibitors of the Severe Acute Respiratory Syndrome Coronavirus (SARS-CoV) 3CL Protease. J. Med. Chem..

[17] Ul Qamar M.T., Alqahtani S.M., Alamri M.A., Chen L.L. (2020). Structural basis of SARS-CoV-2 3CL(pro) and anti-COVID-19 drug discovery from medicinal plants. J. Pharm. Anal..

[18] Berry M., Fielding B., Gamieldien J. (2015). Human coronavirus OC43 3CL protease and the potential of ML188 as a broad-spectrum lead compound: Homology modelling and molecular dynamic studies. BMC Struct. Biol..

[19] Mesecar A.D. (2020). A taxonomically-driven approach to development of potent, broad-spectrum inhibitors of coronavirus main protease including SARS-CoV-2 (COVID-19).

[20] Douangamath A., Fearon D., Gehrtz P., Krojer T., Lukacik P., Owen C.D., Resnick E., Strain-Damerell C., Aimon A., Ábrányi-Balogh P. (2020). Crystallographic and electrophilic fragment screening of the SARS-CoV-2 main protease. Nat. Commun..

[21] Koes D.R., Camacho C.J. (2012). ZINCPharmer: Pharmacophore search of the ZINC database. Nucleic Acids Res..

[22] Trott O., Olson A.J. (2009). AutoDock Vina: Improving the speed and accuracy of docking with a new scoring function, efficient optimization, and multithreading. J. Comput. Chem..

[23] Morris G.M., Huey R., Lindstrom W., Sanner M.F., Belew R.K., Goodsell D.S., Olson A.J. (2009). AutoDock4 and AutoDockTools4: Automated docking with selective receptor flexibility. J. Comput. Chem..

[24] Huey R., Morris G.M., Olson A.J., Goodsell D.S. (2007). A semiempirical free energy force field with charge-based desolvation. J. Comput. Chem..

[25] Grosdidier A., Zoete V., Michielin O. (2011). Fast docking using the CHARMM force field with EADock DSS. J. Comput. Chem..

[26] Jones G.H., Willett P., Glen R.C., Leach A.R., Taylor R. (1997). Development and validation of a genetic algorithm for flexible docking 1 Edited by F. E. Cohen. J. Mol. Biol..

[27] Ding Y., Fang Y., Moreno J., Ramanujam J., Jarrell M., Brylinski M. (2016). Assessing the similarity of ligand binding conformations with the Contact Mode Score. Comput. Biol. Chem..

[28] Alexander S.P., Ball J.K., Tsoleridis T. (2020). Coronavirus (CoV) proteins (version 2020.5) in the IUPHAR/BPS Guide to Pharmacology Database. IUPHAR/BPS Guide Pharmacol. CITE.

[29] PostEra COVID Moonshot. https://covid.postera.ai/covid.

[30] Mendez D., Gaulton A., Bento A.P., Chambers J., De Veij M., Félix E., Magariños M.P., Mosquera J.F., Mutowo P., Nowotka M. (2019). ChEMBL: Towards direct deposition of bioassay data. Nucleic Acids Res..

[31] Ryu Y.B., Jeong H.J., Kim J.H., Kim Y.M., Park J.Y., Kim D., Nguyen T.T., Park S.J., Chang J.S., Park K.H. (2010). Biflavonoids from *Torreya nucifera* displaying SARS-CoV 3CL(pro) inhibition. Bioorg. Med. Chem..

[32] Daina A., Michielin O., Zoete V. (2017). SwissADME: A free web tool to evaluate pharmacokinetics, drug-likeness and medicinal chemistry friendliness of small molecules. Sci. Rep..

[33] Moriguchi I., Hirono S., Liu Q., Nakagome I., Matsushita Y. (1992). Simple Method of Calculating Octanol/Water Partition Coefficient. Chem. Pharm. Bull..

[34] Baell J.B., Nissink J.W.M. (2018). Seven Year Itch: Pan-Assay Interference Compounds (PAINS) in 2017—Utility and Limitations. ACS Chem. Biol..

[35] Daina A., Zoete V. (2016). A BOILED-Egg to Predict Gastrointestinal Absorption and Brain Penetration of Small Molecules. ChemMedChem.

[36] Ali J., Camilleri P., Brown M.B., Hutt A.J., Kirton S.B. (2012). Revisiting the General Solubility Equation: In Silico Prediction of Aqueous Solubility Incorporating the Effect of Topographical Polar Surface Area. J. Chem. Inf. Model..

[37] Daina A., Michielin O., Zoete V. (2014). iLOGP: A Simple, Robust, and Efficient Description of n-Octanol/Water Partition Coefficient for Drug Design Using the GB/SA Approach. J. Chem. Inf. Model..

[38] Chodera J.D., Lee A.A., London N., Von Delft F. (2020). Crowdsourcing drug discovery for pandemics. Nat. Chem..

[39] Li Q., Kang C. (2020). Progress in Developing Inhibitors of SARS-CoV-2 3C-Like Protease. Microorganisms.

[40] Zhou Y., Hou Y., Shen J., Huang Y., Martin W., Cheng F. (2020). Network-based drug repurposing for novel coronavirus 2019-nCoV/SARS-CoV-2. Cell Discov..

[41] Xu C., Ke Z., Liu C., Wang Z., Liu D., Zhang L., Wang J., He W., Xu Z., Li Y. (2020). Systemic in Silico Screening in Drug Discovery for Coronavirus Disease (COVID-19) with an Online Interactive Web Server. J. Chem. Inf. Model..

[42] Carlson E.E. (2010). Natural Products as Chemical Probes. ACS Chem. Biol..

[43] Newman D.J., Cragg G.M. (2020). Natural Products as Sources of New Drugs over the Nearly Four Decades from 01/1981 to 09/2019. J. Nat. Prod..

[44] Rana S., Sharma S., Ghosh K. (2020). Virtual Screening of Naturally Occuring Antiviral Molecules for SARS-CoV-2 Mitigation Using Docking Tool on Multiple Molecular Targets. ChemRxiv.

[45] Riva L., Yuan S., Yin X., Martin-Sancho L., Matsunaga N., Pache L., Burgstaller-Muehlbacher S., De Jesus P.D., Teriete P., Hull M.V. (2020). Discovery of SARS-CoV-2 antiviral drugs through large-scale compound repurposing. Nat. Cell Biol..

[46] Sharma P., Vijayan V., Pant P., Sharma M., Vikram N., Kaur P., Singh T.P., Sharma S. (2020). Identification of potential drug candidates to combat COVID-19: A structural study using the main protease (mpro) of SARS-CoV-2. J. Biomol. Struct. Dyn..

[47] Kandeel M., Al-Nazawi M. (2020). Virtual screening and repurposing of FDA approved drugs against COVID-19 main protease. Life Sci..

[48] Kumar Y., Singh H., Patel C.N. (2020). In silico prediction of potential inhibitors for the main protease of SARS-CoV-2 using molecular docking and dynamics simulation based drug-repurposing. J. Infect. Public Health.

[49] Lokhande K.B., Doiphode S., Vyas R., Swamy K.V. (2020). Molecular docking and simulation studies on SARS-CoV-2 Mpro reveals Mitoxantrone, Leucovorin, Birinapant, and Dynasore as potent drugs against COVID-19. J. Biomol. Struct. Dyn..

[50] Al-Khafaji K., Al-Duhaidahawi D.L., Tok T.T. (2020). Using integrated computational approaches to identify safe and rapid treatment for SARS-CoV-2. J. Biomol. Struct. Dyn..

[51] Mahanta S., Chowdhury P., Gogoi N., Goswami N., Borah D., Kumar R., Chetia D., Borah P., Buragohain A.K., Gogoi B. (2020). Potential anti-viral activity of approved repurposed drug against main protease of SARS-CoV-2: An in silico based approach. J. Biomol. Struct. Dyn..

[52] Shamsi A., Mohammad T., Anwar S., Alajmi M.F., Hussain A., Rehman T., Islam A., Hassan I. (2020). Glecaprevir and Maraviroc are high-affinity inhibitors of SARS-CoV-2 main protease: Possible implication in COVID-19 therapy. Biosci. Rep..

[53] Hu Y., Stumpfe D., Bajorath J. (2016). Recent Advances in Scaffold Hopping. J. Med. Chem..

[54] Grisoni F., Merk D., Byrne R., Schneider G. (2018). Scaffold-Hopping from Synthetic Drugs by Holistic Molecular Representation. Sci. Rep..

[55] Gimeno A., Mestres-Truyol J., Ojeda-Montes M.J., Macip G., Saldivar-Espinoza B., Cereto-Massagué A., Pujadas G., Garcia-Vallvé S. (2020). Prediction of Novel Inhibitors of the Main Protease (M-pro) of SARS-CoV-2 through Consensus Docking and Drug Reposition. Int. J. Mol. Sci..

[56] Koes D.R., Pabon N.A., Deng X., Phillips M.A., Camacho C.J. (2015). A Teach-Discover-Treat Application of ZincPharmer: An Online Interactive Pharmacophore Modeling and Virtual Screening Tool. PLoS ONE.

[57] El Kerdawy A.M., Osman A.A., Zaater M. (2019). Receptor-based pharmacophore modeling, virtual screening, and molecular docking studies for the discovery of novel GSK-3β inhibitors. J. Mol. Model..

[58] Agrawal R., Jain P., Dikshit S.N. (2012). Ligand-based pharmacophore detection, screening of potential gliptins and docking studies to get effective antidiabetic agents. Comb. Chem. High Throughput Screen..

[59] Katarkar A., Haldar P.K., Chaudhuri K. (2015). De novo design based pharmacophore query generation and virtual screening for the discovery of Hsp-47 inhibitors. Biochem. Biophys. Res. Commun..

[60] Salentin S., Schreiber S., Haupt V.J., Adasme M.F., Schroeder M. (2015). PLIP: Fully automated protein–ligand interaction profiler. Nucleic Acids Res..

[61] Laskowski R.A., Swindells M.B. (2011). LigPlot+: Multiple Ligand–Protein Interaction Diagrams for Drug Discovery. J. Chem. Inf. Model..

[62] Yoshino R., Yasuo N., Sekijima M. (2020). Identification of key interactions between SARS-CoV-2 main protease and inhibitor drug candidates. Sci. Rep..

[63] Chen P., Ke Y., Lu Y., Du Y., Li J., Yan H., Zhao H., Zhou Y., Yang Y. (2019). DLIGAND2: An improved knowledge-based energy function for protein–ligand interactions using the distance-scaled, finite, ideal-gas reference state. J. Cheminform..

[64] Theerawatanasirikul S., Kuo C.J., Phetcharat N., Lekcharoensuk P. (2020). In silico and in vitro analysis of small molecules and natural compounds targeting the 3CL protease of feline infectious peritonitis virus. Antivir. Res..

[65] Jeong J., Kim H., Choi J. (2019). In Silico Molecular Docking and In Vivo Validation with Caenorhabditis elegans to Discover Molecular Initiating Events in Adverse Outcome Pathway Framework: Case Study on Endocrine-Disrupting Chemicals with Estrogen and Androgen Receptors. Int. J. Mol. Sci..

[66] Cuccioloni M., Bonfili L., Cecarini V., Cocchioni F., Petrelli D., Crotti E., Zanchi R., Eleuteri A.M., Angeletti M. (2020). Structure/activity virtual screening and in vitro testing of small molecule inhibitors of 8-hydroxy-5-deazaflavin:NADPH oxidoreductase from gut methanogenic bacteria. Sci. Rep..

[67] Irwin J.J., Shoichet B.K. (2005). ZINC-a free database of commercially available compounds for virtual screening. J. Chem. Inf. Model..

[68] Abagyan R., Totrov M., Kuznetsov D. (1994). ICM A new method for protein modeling and design: Applications to docking and structure prediction from the distorted native conformation. J. Comput. Chem..

[69] Callejo G., Pattison L.A., Greenhalgh J.C., Chakrabarti S., Andreopoulou E., Hockley J.R., Smith E.S.J., Rahman T. (2020). In silico screening of GMQ-like compounds reveals guanabenz and sephin1 as new allosteric modulators of acid-sensing ion channel 3. Biochem. Pharmacol..

[70] Greenhalgh J.C., Chandran A., Harper M.T., Ladds G., Rahman T. (2020). Proposed model of the Dictyostelium cAMP receptors bound to cAMP. J. Mol. Graph. Model..

[71] Bell E.W., Zhang Y. (2019). DockRMSD: An open-source tool for atom mapping and RMSD calculation of symmetric molecules through graph isomorphism. J. Cheminform..

[72] Pettersen E.F., Goddard T.D., Huang C.C., Couch G.S., Greenblatt D.M., Meng E.C., Ferrin T.E. (2004). UCSF Chimera-A visualization system for exploratory research and analysis. J. Comput. Chem..

[73] R Core Team (2016). R: A Language and Environment for Statistical Computing.

[74] ChemAxon (2020). Marvin, 20.16.

