# Supplementary material for: Novel Small-Molecule Scaffolds as Candidates against the SARS Coronavirus 2 Main Protease: A Fragment-Guided in Silico Approach
Source: Molecules. 2020 Nov 24;25(23):5501. doi: 10.3390/molecules25235501 (PMC7727661; doi:10.3390/molecules25235501)
Supplement: Supplementary file 1 [file molecules-25-05501-s001.pdf]

Novel Small Molecule Scaffolds as Candidates Against the SARS Coronavirus 2 Main Protease: a Fragment-Guided *In Silico* Approach

SUPPLEMENTAL MATERIAL

Teresa L. Augustin‡, Roxanna Hajbabaie‡, Matthew T. Harper, Taufiq Rahman**

‡These authors contributed equally to this work and should be considered as co-first authors.

Department of Pharmacology, University of Cambridge, Tennis Court Road, Cambridge CB2 1PD, UK

** Corresponding Author
Taufiq Rahman: mtur2@cam.ac.uk

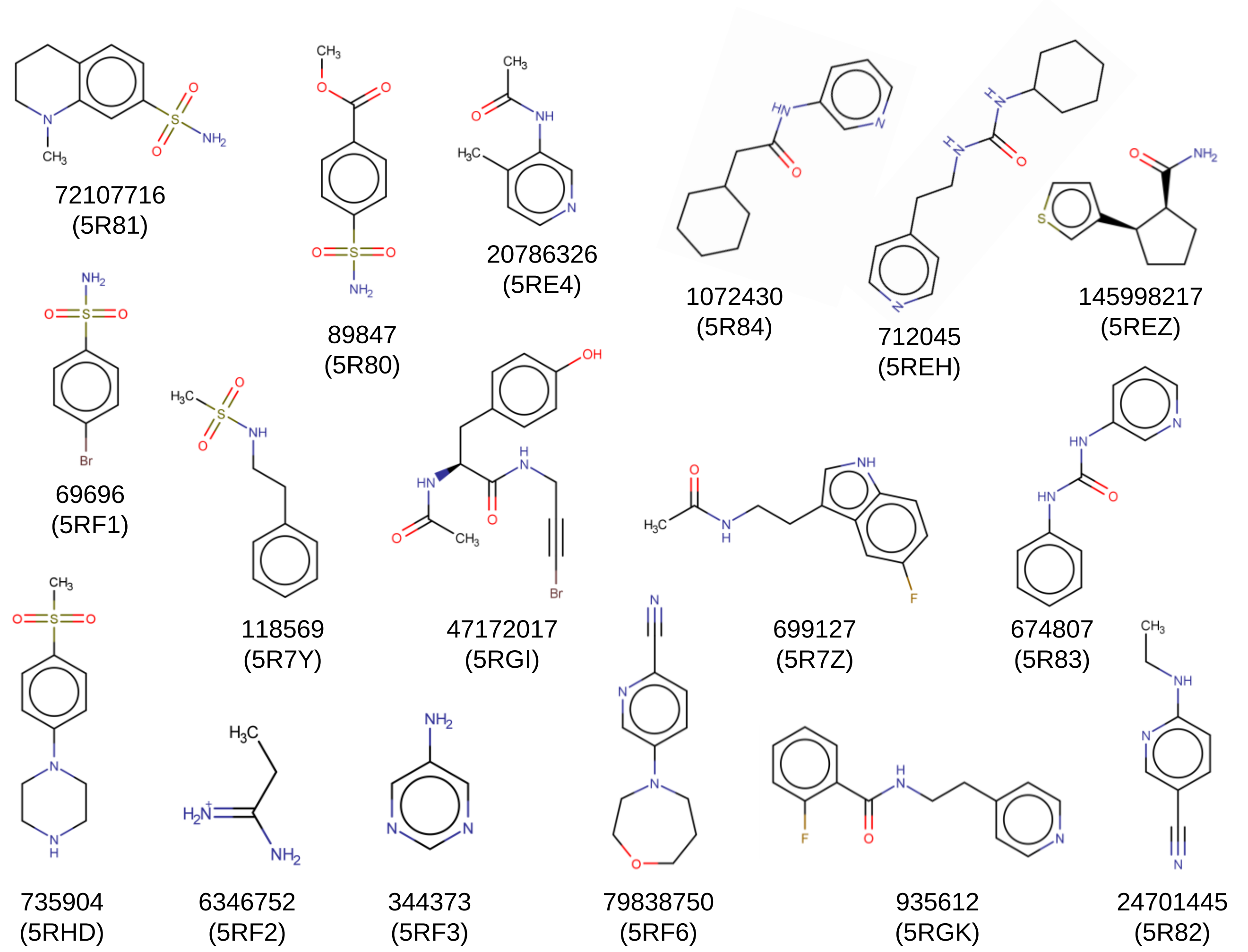

Figure S1: 2D structures of the 17 active fragments from the SARS-CoV-2 M^{pro} XChem screen. The fragment PubChem CIDs are shown, and the PDB IDs are in parentheses.

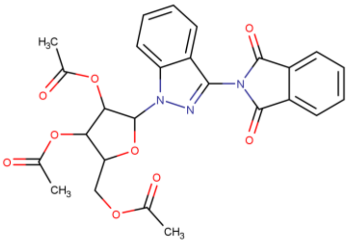

7173849

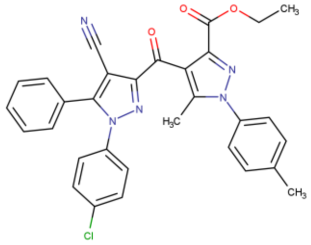

2754601

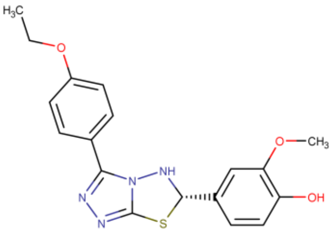

7052206

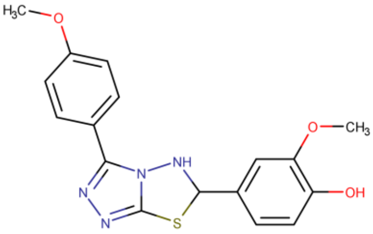

2747802

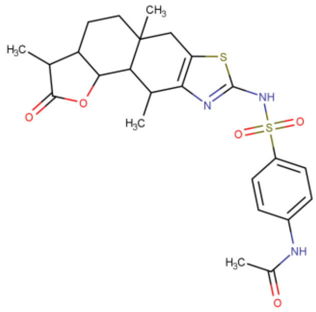

11866245

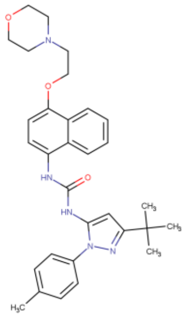

156422

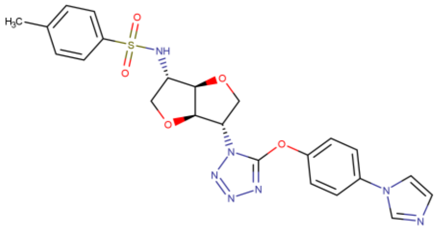

40474970

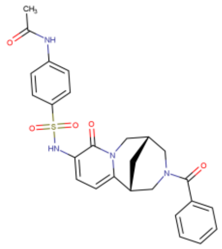

11867146

Figure S2: 2D structures of the remaining compounds from focused docking (those not shown in Figure 3). Compound labels are PubChem CIDs.

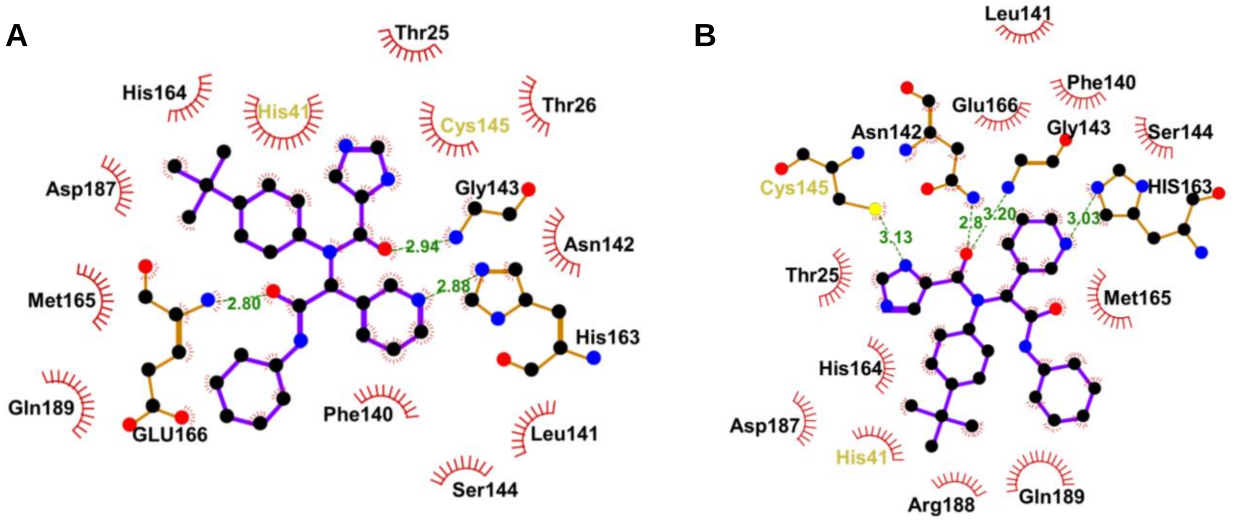

Figure S3: Predicted interactions in LigPlot+ of the original co-crystallized X77 ligand (purple) with the SARS-CoV-2 M^{pro} (PDB ID: 6W63). **A)** Original co-crystallized X77 pose. **B)** X77 pose from AutoDock Vina. Hydrogen bonds (green) shown with distance between the ligand and brown residues. Hydrophobic interactions with residues marked in red. The catalytic dyad, His41 and Cys145, are highlighted in gold.

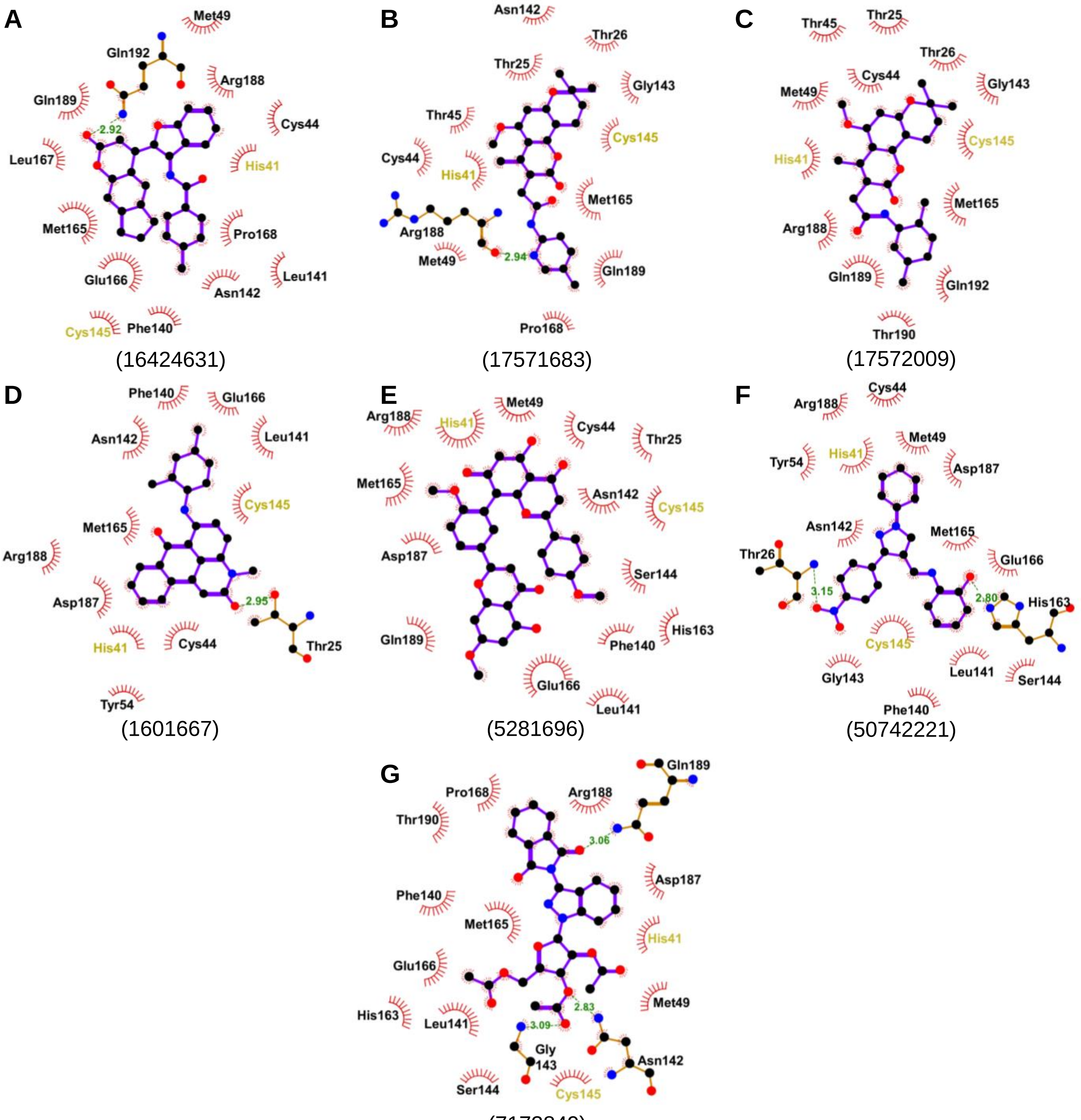

Figure S4 (A-G): Predicted interactions in LigPlot^{+} of the top seven selected compounds (purple) with the SARS-CoV-2 M^{pro} (PDB ID: 6W63). Hydrogen bonds (green) shown with distance between the ligand and brown residues. Hydrophobic interactions with residues marked in red. The catalytic dyad, His41 and Cys145, are highlighted in gold. Compound labels are PubChem CIDs.

Table S1: Blind docking scores from AutoDock Vina for all 134 selected compounds from ZINCPharmer and the X77 control. Comments indicate whether the given AutoDock Vina score for the active site was not the most negative score.

PubChem CID	AutoDock Vina binding affinity (kcal/mol)	Comment
17571683	-9.4	
1601667	-9.2	
16424631	-9.1	
17572009	-9.1	
5281696	-9.1	
50742221	-8.8	
7173849	-8.8	
2754601	-8.7	2nd score
145998279 (X77)	-8.7	
156422	-8.6	
40474970	-8.6	
11866245	-8.5	
7173228	-8.5	
11867146	-8.5	
2747802	-8.5	
7052206	-8.5	
18568846	-8.3	
1790912	-8.2	
18568635	-8.2	
1752298	-8.2	
133556351	-8.2	
40826705	-8.1	
136868522	-8.1	2nd score
16433229	-8.1	
4586871	-8.1	2nd score
8016335	-8	2nd score
5704794	-8	
52905552	-8	
135582848	-8	
1609365	-8	
6224678	-7.9	
5530142	-7.9	
2320869	-7.9	2nd score
40777541	-7.9	
24280197	-7.8	
52905269	-7.8	
662650	-7.8	
7052113	-7.8	2nd score
71692218	-7.7	
16433230	-7.7	
2828649	-7.7	
86308329	-7.6	
11865521	-7.6	
16403518	-7.6	
104850	-7.6	2nd score

Table S1 (continued)

PubChem CID	AutoDock Vina binding affinity (kcal/mol)	Comment
71753578	-7.6	
939120	-7.6	
16396750	-7.5	2nd score
135651551	-7.5	3rd score
17572052	-7.5	
1773531	-7.5	
788023	-7.5	
342450	-7.4	
40780421	-7.4	
9362786	-7.4	
3648622	-7.4	
52905125	-7.4	
124246	-7.3	
1745243	-7.3	
42648976	-7.3	
17572026	-7.3	
62977	-7.3	
99613	-7.3	
16405987	-7.3	3rd score
100640	-7.3	
78237	-7.3	
51722607	-7.2	
404564	-7.2	
1799898	-7.2	
10688053	-7.2	2nd score
249422	-7.2	
2755751	-7.2	4th score
3220	-7.2	
56723758	-7.1	
26194061	-7.1	2nd score
9476	-7.1	
24281188	-7.1	
905132	-7.1	
6236017	-7.1	
124211	-7	
39063552	-7	2nd score
5271991	-7	
2749877	-7	
10937587	-7	
2800908	-7	
6662	-6.9	
4670964	-6.9	
160743	-6.9	
40777546	-6.8	
38353074	-6.8	
2748632	-6.8	

Table S1 (continued)

PubChem CID	AutoDock Vina binding affinity (kcal/mol)	Comment
16409266	-6.7	
50741908	-6.7	
65096	-6.7	
308397	-6.7	
25755594	-6.7	2nd score
2749243	-6.6	
47979	-6.6	
68213	-6.6	
6911915	-6.5	2nd score
695972	-6.5	
3276884	-6.4	
5353453	-6.4	3rd score
2747917	-6.4	
13086	-6.4	2nd score
38854	-6.4	
2749693	-6.3	
2754050	-6.3	
2787097	-6.3	
6921661	-6.3	
42648684	-6.3	3rd score
136048	-6.3	3rd score
5394804	-6.2	
26116438	-6.1	
1761763	-6	
594973	-6	
7021	-6	
2020836	-5.9	
11886099	-5.9	
38853	-5.7	
12241315	-5.7	
42261216	-5.6	
49384	-5.4	
135398733	-5.4	
22784	-5.4	3rd score
5233029	-5.4	2nd score
30479	-5.4	
1986	-5.3	2nd score
70789	-5.2	2nd score
37256	-5.1	
391402	-5.1	5th score
560394	-4.3	2nd score
5706223	-3.8	7th score
7311729	-3.3	4th score

Table S2: List of vendors with IDs and SMILES strings of the 26 compounds included from focused docking (AutoDock Vina).

PubChem CID (also known as)	Vendor	Vendor ID	SMILES
**16424631**	MolPort	MolPort-000-857-054	CC1=CC=C(C=C1)C(=O)NC2=C(OC3=CC=CC=C32)C4=CC(=O)OC5=C4C=C6CCCC6=C5
**17571683**	MolPort	MolPort-005-910-104	CC1=CN=C(C=C1)NC(=O)CC2=C(C3=C(C=C4C(=C3OC2=O)CCC(O4)(C)C)OC)C
**17572009**	MolPort	MolPort-005-910-434	CC1=CC(=C(C=C1)C)NC(=O)CC2=C(C3=C(C=C4C(=C3OC2=O)CCC(O4)(C)C)OC)C
**1601667**	MolPort	MolPort-000-644-441	CC1=CC(=C(C=C1)NC2=C3C4=C(C=C2)N(C(=O)C=C4C5=CC=CC=C5C3=O)C)C
**50742221**	MolMall	21088	C1=CC=C(C=C1)N2C=C(C(=N2)C3=CC=C(C=C3)[N+](=O)[O-])C=NC4=CC=CC=C4O
**5281696 (sciadopitysin)**	MolPort	MolPort-009-754-970	COC1=CC=C(C=C1)C2=CC(=O)C3=C(O2)C(=C(C=C3O)O)C4=C(C=CC(=C4)C5=CC(=O)C6=C(C=C(C=C6O5)OC)O)OC
**11866245**	MolPort	MolPort-001-750-463	C[C@H]1[C@@H]2CC[C@]3(CC4=C([C@H]([C@@H]3[C@H]2OC1=O)C)N=C(S4)NS(=O)(=O)C5=CC=C(C=C5)NC(=O)C)C
**7173849**	MolMall	20467	CC(=O)OC[C@H]1[C@@H]([C@@H]([C@@H](O1)N2C3=CC=CC=C3C(=N2)N4C(=O)C5=CC=CC=C5C4=O)OC(=O)C)OC(=O)C
**2754601**	MolMall	18880	CCOC(=O)C1=NN(C(=C1C(=O)C2=NN(C(=C2C#N)C3=CC=CC=C3)C4=CC=C(C=C4)Cl)C)C5=CC=C(C=C5)C
**156422 (doramapimod)**	MolPort	MolPort-006-168-795	CC1=CC=C(C=C1)N2C(=CC(=N2)C(C)(C)C)NC(=O)NC3=CC=C(C4=CC=CC=C43)OCCN5CCOCC5
**2747802**	MolMall	567	COC1=CC=C(C=C1)C2=NN=C3N2NC(S3)C4=CC(=C(C=C4)O)OC
**40474970**	MolPort	MolPort-001-751-888	CC1=CC=C(C=C1)S(=O)(=O)N[C@H]2CO[C@H]3[C@@H]2OC[C@@H]3N4C(=NN=N4)OC5=CC=C(C=C5)N6C=CN=C6
**7173228**	MolMall	17800	CCOC(=O)C1=NN(C(=C1C(=O)C2=NO[C@H]3[C@H]2C(=O)N(C3=O)C4=C(C=CC(=C4)Cl)Cl)C)C5=CC=C(C=C5)C
**11867146**	MolPort	MolPort-001-742-862	CC(=O)NC1=CC=C(C=C1)S(=O)(=O)NC2=CC=C3[C@@H]4C[C@@H](CN(C4)C(=O)C5=CC=CC=C5)CN3C2=O
**7052206**	MolMall	617	CCOC1=CC=C(C=C1)C2=NN=C3N2N[C@@H](S3)C4=CC(=C(C=C4)O)OC

Table S3: List of known SARS-CoV-1/SARS-CoV-2 M^{pro} inhibitors for scaffold novelty comparison (related scaffolds highlighted in blue).

PubChem CID (also known as)	Inhibitor of	Source	SMILES
**11561899 (PF-00835231)**	SARS-CoV-1 M^{pro}	IUPHAR/BPS Guide to PHARMACOLOGY	OCC(=O)[C@@H](NC(=O)[C@@H](NC(=O)c1[nH]c2c(c1)c(OC)ccc2)CC(C)C)C[C@@H]1CCNC1=O
**11844232 (TG-0205221)**	SARS-CoV-1 M^{pro}	IUPHAR/BPS Guide to PHARMACOLOGY	OC[C@@H](NC(=O)[C@@H](NC(=O)[C@H]([C@H](OC(C)(C)C)C)NC(=O)OCc1ccccc1)CC1CCCCC1)C[C@@H]1CCNC1=O
**10324367 (boceprevir)**	SARS-CoV-2 M^{pro}	IUPHAR/BPS Guide to PHARMACOLOGY & PostEra	O=C(NC(C)(C)C)N[C@@H](C(C)(C)C)C(=O)N1C[C@H]2[C@@H]([C@H]1C(=O)NC(C(=O)C(=O)N)CC1CCC1)C2(C)C
**71481120 (GC-376)**	SARS-CoV-2 M^{pro}	IUPHAR/BPS Guide to PHARMACOLOGY	CC(C[C@@H](C(=O)N[C@H](C(S(=O)(=O)O)O)CC1CCNC1=O)NC(=O)OCc1ccccc1)C
**25110701 (ALP-POS-c59291d4-5)**	SARS-CoV-1 and SARS-CoV-2 M^{pro}s	IUPHAR/BPS Guide to PHARMACOLOGY	Clc1cncc(c1)OC(=O)c1cccc2c1cc[nH]2
**Not found (MP13)**	SARS-CoV-2 M^{pro}	IUPHAR/BPS Guide to PHARMACOLOGY	O=C[C@@H](NC(=O)[C@@H](NC(=O)[C@H](C(C)C)NC(=O)OCc1ccccc1)CC(C)C)C[C@@H]1CCNC1=O
**146672237 (compound 11b)**	SARS-CoV-2 M^{pro}	IUPHAR/BPS Guide to PHARMACOLOGY & PostEra	O=C[C@@H](C[C@@H]1CCNC1=O)NC(=O)[C@@H](NC(=O)c1cc2c([nH]1)cccc2)Cc1cccc(c1)F
**145343771 (compound 11a)**	SARS-CoV-2 M^{pro}	IUPHAR/BPS Guide to PHARMACOLOGY & PostEra	O=C[C@H](C[C@@H]1CCNC1=O)NC(=O)[C@@H](NC(=O)c1cc2c([nH]1)cccc2)CC1CCCCC1
**Not found (compound 6e)**	SARS-CoV-1 and SARS-CoV-2 M^{pro}s	IUPHAR/BPS Guide to PHARMACOLOGY	CCCCC1CCC(CC1)OC(=O)N[C@H](C(=O)N[C@@H](C[C@@H]1CCNC1=O)C=O)CC(C)C
**145996541 (compound 11r)**	SARS-CoV-1 and SARS-CoV-2 M^{pro}s	IUPHAR/BPS Guide to PHARMACOLOGY	O=C(N[C@H](C(=O)N[C@H](C(=O)C(=O)NCc1ccccc1)C[C@@H]1CCNC1=O)CC1CCCCC1)/C=C/c1ccccc1
**3164070 (walrycin B)**	SARS-CoV-2 M^{pro}	IUPHAR/BPS Guide to PHARMACOLOGY	Cn1nc(nc2c1nc(=O)n(c2=O)C)c1ccc(cc1)C(F)(F)F
**154699454 (DAV-CRI-14a23e73-1)**	SARS-CoV-2 M^{pro}	IUPHAR/BPS Guide to PHARMACOLOGY	ClCC(=O)NC(c1cc(Cl)cc(c1)c1ccc(cc1)S(=O)(=O)N)C
**Not found (compound 6j)**	SARS-CoV-1 and SARS-CoV-2 M^{pro}s	IUPHAR/BPS Guide to PHARMACOLOGY	O=C[C@@H](NC(=O)[C@@H](NC(=O)OCC1CCC(CC1)(F)F)CC(C)C)C[C@@H]1CCNC1=O
**146018708 (compound 13b)**	SARS-CoV-2 M^{pro}	IUPHAR/BPS Guide to PHARMACOLOGY & PostEra	O=C(OC(C)(C)C)Nc1cccn(c1=O)[C@H](C(=O)N[C@H]([C@H](C(=O)NCc1ccccc1)O)C[C@@H]1CCNC1=O)CC1CC1

Table S3 (continued)

PubChem CID (also known as)	Inhibitor of	Source	SMILES
**146025593 (PRD_002214, also N3)**	SARS-CoV-2 M^{pro}	IUPHAR/BPS Guide to PHARMACOLOGY & PostEra	CCCCC1CCC(CC1)OC(=O)N[C@H](C(=O)N[C@@H](C[C@@H]1CCNC1=O)C=O)CC(C)C
**6915837 (Z-FA-FMK)**	SARS-CoV-2 M^{pro}	IUPHAR/BPS Guide to PHARMACOLOGY	FCC(=O)C(NC(=O)[C@H](Cc1ccccc1)NC(=O)OCc1ccccc1)C
**5281605 (baicalein)**	SARS-CoV-2 M^{pro}	PostEra	Oc1cc2OC(=CC(=O)c2c(O)c1O)c3ccccc3
**2577 (carmofur)**	SARS-CoV-2 M^{pro}	PostEra	CCCCCCNC(=O)N1C=C(C(=O)NC1=O)F
**146027054 (pyrithione zinc)**	SARS-CoV-2 M^{pro}	PostEra	C1=CC(=S)N(C=C1)[O-].C1=CC(=S)N(C=C1)[O-].[Zn+2]
**145998279 (X77)**	SARS-CoV-2 M^{pro}	PostEra	CC(C)(C)c1ccc(cc1)N([C@@H](C(=O)NC2CCCCC2)c3cccnc3)C(=O)c4c[nH]cn4
**146018711**	SARS-CoV-2 M^{pro}	PostEra	CC(C)(C)OC(=O)NC1=CC=CN(C1=O)[C@@H](CC2CCCCC2)C(=O)N[C@@H](C[C@@H]3CC=NC3=O)[C@H](C(=O)NC4CC4)O
**2799606**	SARS-CoV-1 M^{pro}	PostEra	Cc1cc(c(Cl)cc1Cl)[S](=O)(=O)c2c(cc(cc2[N+]([O-])=O)C(F)(F)F)[N+]([O-])=O
**2822496**	SARS-CoV-1 M^{pro}	PostEra	FC(F)(F)c1[nH]c(SC(=O)c2oc(cc2)C#Cc3ccccc3)nn1
**25681649**	SARS-CoV-1 M^{pro}	PostEra	CCC(C)(C)NC(=O)[C@H](N(C(=O)Cn1nnc2ccccc12)c3ccc(NC(C)=O)cc3)c4cccn4C
**46897844 (ML188)**	SARS-CoV-1 M^{pro}	PostEra	CC(C)(C)NC(=O)[C@H](N(C(=O)c1occc1)c2ccc(cc2)C(C)(C)C)c3cccnc3
**5287569 (N9)**	SARS-CoV-1 M^{pro}	PostEra	CCOC(=O)/C=C/[C@H](C[C@@H]1CCNC1=O)NC(=O)[C@H](CC(C)C)NC(=O)[C@@H](NC(=O)[C@@H](NC(=O)c2cocc2)C(C)C)C(C)C
**10062715**	SARS-CoV-1 M^{pro}	PostEra	CCOC(=O)/C=C/[C@H](C[C@@H]1CCNC1=O)NC(=O)[C@H](CC=C(C)C)CC(=O)[C@@H](NC(=O)c2cc(C)on2)C(C)C

Table S3 (continued)

PubChem CID (also known as)	Inhibitor of	Source	SMILES
**5287723**	SARS-CoV-1 M^{pro}	PostEra	CCOC(=O)[C@@H](O)CC(=O)N(CCC(N)=O)NC(=O)[C@H](Cc1ccccc1)NC(=O)[C@H](CC(C)C)NC(=O)OCc2ccccc2
**11844232**	SARS-CoV-1 M^{pro}	PostEra	C[C@@H](OC(C)(C)C)[C@H](NC(=O)OCc1ccccc1)C(=O)N[C@@H](CC2CCCCC2)C(=O)N[C@H](CO)C[C@@H]3CCNC3=O
**16220131**	SARS-CoV-1 M^{pro}	PostEra	C[C@@H](NC(=O)[C@H](Cc1ccccc1)NC(=O)OCc2ccccc2)C(=O)CCO
**49866386**	SARS-CoV-1 M^{pro}	PostEra	CC(C)C[C@H](NC(=O)[C@@H](NC(=O)[C@@H](NC(C)=O)C(C)C)[C@@H](C)OCc1ccccc1)C(=O)N[C@@H](C[C@@H]2CCNC2=O)[C@@H](C)O
**12092**	SARS-CoV-1 M^{pro}	PostEra	CN(C)C1=CC=C(C=C1)C(=O)O
